# Digital Tracking of Physical Activity, Heart Rate, and Inhalation Behavior in Patients With Pulmonary Arterial Hypertension Treated With Inhaled Iloprost: Observational Study (VENTASTEP)

**DOI:** 10.2196/25163

**Published:** 2021-10-08

**Authors:** Barbara Stollfuss, Manuel Richter, Daniel Drömann, Hans Klose, Martin Schwaiblmair, Ekkehard Gruenig, Ralf Ewert, Martin C Kirchner, Frank Kleinjung, Valeska Irrgang, Christian Mueller

**Affiliations:** 1 Bayer Vital GmbH Leverkusen Germany; 2 Department of Internal Medicine Justus-Liebig-University Giessen, Universities of Giessen and Marburg Lung Center Member of the German Center for Lung Research Giessen Germany; 3 Department of Pneumology Universitätsklinikum Schleswig-Holstein Member of the German Center for Lung Research Lübeck Germany; 4 Department of Pneumology Universitätsklinikum Hamburg-Eppendorf Hamburg Germany; 5 Department of Pneumology I. Medizinische Klinik Universitätsklinikum Augsburg Augsburg Germany; 6 Centre for Pulmonary Hypertension Thoraxclinic Heidelberg GmbH at Heidelberg University Hospital and German Centre for Lung Research Heidelberg Germany; 7 Department of Internal Medicine Universitätsmedizin Greifswald Greifswald Germany; 8 Bayer AG Berlin Germany

**Keywords:** 6-minute walk distance, 6MWD, Breelib, daily physical activity, digital monitoring, health-related quality of life, iloprost, Ventavis, inhalation behavior, mobile phone, pulmonary arterial hypertension, PAH, sleeping behavior, behavior, sleep, monitoring, physical activity, heart, cardiology

## Abstract

**Background:**

Pulmonary arterial hypertension restricts the ability of patients to perform routine physical activities. As part of pulmonary arterial hypertension treatment, inhaled iloprost can be administered via a nebulizer that tracks inhalation behavior. Pulmonary arterial hypertension treatment is guided by intermittent clinical measurements, such as 6-minute walk distance, assessed during regular physician visits. Continuous digital monitoring of physical activity may facilitate more complete assessment of the impact of pulmonary arterial hypertension on daily life. Physical activity tracking with a wearable has not yet been assessed with simultaneous tracking of pulmonary arterial hypertension medication intake.

**Objective:**

We aimed to digitally track the physical parameters of patients with pulmonary arterial hypertension who were starting treatment with iloprost using a Breelib nebulizer. The primary objective was to investigate correlations between changes in digital physical activity measures and changes in traditional clinical measures and health-related quality of life over 3 months. Secondary objectives were to evaluate inhalation behavior, adverse events, and changes in heart rate and sleep quality.

**Methods:**

We conducted a prospective, multicenter observational study of adults with pulmonary arterial hypertension in World Health Organization functional class III who were adding inhaled iloprost to existing pulmonary arterial hypertension therapy. Daily distance walked, step count, number of standing-up events, heart rate, and 6-minute walk distance were digitally captured using smartwatch (Apple Watch Series 2) and smartphone (iPhone 6S) apps during a 3-month observation period (which began when iloprost treatment began). Before and at the end of the observation period (within 2 weeks), we also evaluated 6-minute walk distance, Borg dyspnea, functional class, B-type natriuretic peptide (or N-terminal pro–B-type natriuretic peptide) levels, health-related quality of life (EQ-5D questionnaire), and sleep quality (Pittsburgh Sleep Quality Index).

**Results:**

Of 31 patients, 18 were included in the full analysis (observation period: median 91.5 days, IQR 88.0 to 92.0). Changes from baseline in traditional and digital 6-minute walk distance were moderately correlated (r=0.57). Physical activity (daily distance walked: median 0.4 km, IQR –0.2 to 1.9; daily step count: median 591, IQR −509 to 2413) and clinical measures (traditional 6-minute walk distance: median 26 m, IQR 0 to 40) changed concordantly from baseline to the end of the observation period. Health-related quality of life showed little change. Total sleep score and resting heart rate slightly decreased. Distance walked and step count showed short-term increases after each iloprost inhalation. No new safety signals were identified (safety analysis set: n=30).

**Conclusions:**

Our results suggest that despite challenges, parallel monitoring of physical activity, heart rate, and iloprost inhalation is feasible in patients with pulmonary arterial hypertension and may complement traditional measures in guiding treatment; however, the sample size of this study limits generalizability.

**Trial Registration:**

ClinicalTrials.gov NCT03293407; https://clinicaltrials.gov/ct2/show/NCT03293407

**International Registered Report Identifier (IRRID):**

RR2-10.2196/12144

## Introduction

### Background

Remote patient monitoring can play an important role in disease management, supporting the conventional approach of face-to-face visits. In patients with chronic diseases such as left-sided heart failure, remote monitoring has been reported to have beneficial effects—reduced readmissions (possibly due to earlier treatment for symptoms detected before scheduled follow-up visits), reduced mortality, increased quality of life, increased participation in self-management of the disease, and improved knowledge of the disease [1-4].

Pulmonary arterial hypertension is a progressive, life-threatening disease that can lead to right-sided heart failure and death [5]. Dyspnea, a common symptom of pulmonary arterial hypertension, restricts physical activity, which in turn impairs quality of life. The impairment of quality of life in patients with pulmonary arterial hypertension is severe, with health-related quality of life scores comparable to those reported for patients with spinal cord injuries or cancer unresponsive to therapy [6]. However, the use of vasoactive treatment to address pulmonary arterial hypertension is usually guided by intermittent clinical measurements of the 6-minute walk distance (a routine assessment of exercise capacity in pulmonary arterial hypertension), World Health Organization (WHO) functional class, and levels of B-type natriuretic peptide (BNP) or N-terminal pro–B-type natriuretic peptide (NT-proBNP) [7]. These traditional clinical measures are assessed during regular out- or inpatient visits, usually at widely spaced intervals (eg, every 3-6 months), and therefore, provide only a snapshot of the patient’s health status. In times of increased reliance on telemedicine, such as during the current COVID-19 pandemic, visits between patients and their treating physicians may not be possible. For example, patients may attempt to reduce the probability of infection by avoiding visits in hospitals, and hospitals may enact restrictions and cancel visits. Furthermore, traditional clinical parameters do not address the impact of pulmonary arterial hypertension on daily life, which may be more important to the patient than functional or laboratory measures [8,9]. Remote monitoring may provide a more complete picture by allowing continuous assessment of parameters (such as physical activity) that reflect the impact of pulmonary arterial hypertension on daily life. However, it is unclear whether digital measures of physical activity are associated with traditional clinical parameters during long-term treatment. Furthermore, physical activity has not yet been assessed simultaneously with medication intake tracking.

Iloprost, a prostacyclin-based inhaled therapy for patients with primary pulmonary hypertension (idiopathic pulmonary arterial hypertension) in WHO functional class III, has shown beneficial effects on 6-minute walk distance and WHO functional class in clinical trials [10-12]. Iloprost is administered via nebulizers such as the Breelib nebulizer (Vectura Group plc) [13], which automatically saves inhalation information (dates, times, and completeness of inhalations).

### Objectives

The primary aim of our study was to track daily physical activity in patients with pulmonary arterial hypertension starting treatment with inhaled iloprost, using a commercially available smartwatch and smartphone, and to investigate the association between changes in digital physical activity measures and changes in traditional clinical measures of disease severity and health-related quality of life over a 12-week period.

Secondary aims were to assess acceptance of the smartwatch by patients and the feasibility of measuring 6-minute walk distance digitally and to identify digital measures linked to traditional 6-minute walk distance. Further aims were to digitally monitor heart rate and iloprost inhalation behavior, to explore temporal patterns in activity level and heart rate relative to iloprost inhalation periods, and to evaluate changes in clinical outcome measures, daily physical activity, heart rate, and sleep quality after initiation of inhaled iloprost therapy. The study was not designed to investigate or confirm the effectiveness and safety of iloprost (Ventavis).

## Methods

### Study Design

Additional details are available in a previous publication [14] and in Multimedia Appendix 1.

As part of a national, prospective, observational, multicenter, single-arm cohort study (VENTASTEP; Clinical trial.gov, NCT03293407) investigating the associations between clinical and device-reported outcomes in patients with pulmonary arterial hypertension (WHO functional class III) who were adding inhaled iloprost to existing pulmonary arterial hypertension therapy, we collected digital measures of daily physical activity, heart rate, and 6-minute walk distance using smartwatch (Apple Watch Series 2) and smartphone (Apple iPhone 6S) apps (xbird GmbH). The apps continuously captured data from the smartwatch for the purpose of analyzing physical activity and behavior patterns; the 6-minute walk distance app captured data from the smartwatch during the 6-minute walk distance test. Raw sensor data from the smartphone were not used because it was impossible to ascertain whether patients always carried the smartphone with them. Device functionality was reduced to the minimum requirements for the study. To reduce bias, the apps did not provide feedback on physical activity to patients or physicians. A nebulizer (Breelib, Bayer AG) was used to record inhalation behavior [13,15,16].

The study period consisted of baseline data collection during the period from the initial visit and decision to start iloprost treatment until the actual start of iloprost treatment (≤2 weeks), an observation period of 3 months (±2 weeks) from the start of iloprost treatment, and final data collection at discontinuation of therapy or at the end of the study (whichever was earliest). The decision to start treatment with inhaled iloprost was made at the discretion of the treating physician; the decision was made in advance and independently of inclusion in the study. Follow-up was conducted during routine patient visits to their pulmonary arterial hypertension centers.

The study was approved by the central ethics committee of the Justus Liebig University Giessen (AZ153/17) and the ethics committees of all participating sites.

### Patients

In preparation for this study, a survey on acceptance of digital tracking was performed, which indicated that approximately 50% of patients were willing to participate [14].

Patients were enrolled from 7 specialized pulmonary arterial hypertension centers in Germany from February 2018 to July 2019. Patients aged ≥18 years with pulmonary arterial hypertension in WHO functional class III despite treatment were eligible for enrollment if they were willing to wear a smartwatch for the duration of the study, had no previous treatment with inhaled iloprost, and they and their treating physician had decided to initiate treatment with inhaled iloprost using a Breelib nebulizer. Patients were excluded if they were allergic to nickel and methacrylates, or if they were already participating in an investigational program that included an intervention outside of routine clinical practice. All patients who were enrolled provided signed informed consent.

Study participants were trained by site personnel or investigators to correctly use the smartphone and smartwatch. Study participants were given training on inhaler use through a patient support program (VENTAPLUS, ContraCare GmbH and Vitartis Medical-Services GmbH), which is routinely offered to patients.

### Data Collection

Anamnestic data (demographic and clinical characteristics) were collected by an investigator from medical records, if available, and entered into an electronic case report form that formed part of an electronic data capture system developed and managed by a contract research organization (Institut Dr. Schauerte).

Traditional clinical measures (6-minute walk distance, Borg dyspnea score at end of 6-minute walk distance test, WHO functional class, and levels of BNP or NT-proBNP) and vital parameters were assessed at the initial and final visit to the study center; data were also recorded in the electronic case report form. Patient-reported outcomes—EuroQol 5-dimension questionnaire (EQ-5D) and Pittsburgh Sleep Quality Index (PSQI)—were documented on paper at the initial and final visits and sent to the contract research organization for analysis. Adverse events, from the periods between the first use of inhaled iloprost to 30 days after the last dose within the study period, were documented in the electronic case report form.

Digital measurements of daily physical activity and heart rate were captured continuously by the smartwatch and app. Digital 6-minute walk distance data (steps, distance walked, heart rate, and raw data from motion sensors) were collected at the initial and final visit using the smartwatch and 6-minute walk distance app (which uses step count and a stride length algorithm trained on healthy volunteer data). Inhalation data (frequency of inhalations, completeness of inhalations, and duration of inhalations) were captured by the nebulizer, transferred to the smartphone using the BreeConnect App (Bayer AG), and then transferred to the contract research organization for analysis.

All data collected by the smartwatch were stored pseudonymized on a secure cloud server (xbird GmbH) for further processing. Processed data points for all variables were transferred as pseudonymized data to the electronic data capture system and matched to other collected data after the last visit of the last patient.

### Outcome Measures

The primary endpoint was correlation between changes from baseline to the final visit in digital measures of physical activity, traditional clinical measures, and in health-related quality of life. Changes from baseline were evaluated as a first step. The traditional clinical measures included in the primary analysis were the 6-minute walk distance, Borg dyspnea score after the 6-minute walk distance test, WHO functional class, and BNP or NT-proBNP levels. Health-related quality of life was assessed using the EQ-5D. The digital measures were distance walked, number of steps, number of standing-up events, and digital 6-minute walk distance (Multimedia Appendix 2).

Secondary endpoints included inhalation behavior (mean daily inhalation duration per session, mean daily number of inhalations, and mean daily proportion of complete and incomplete inhalations) and the mean association between physical activity level (device-based) and time since the last inhalation. The change from baseline to the end of the observation period in sleep quality (PSQI) and heart rate (at rest and during the 6-minute walk distance test), the association between heart rate and other device-based measures during the whole study period, and the incidence of treatment-emergent adverse events and serious treatment-emergent adverse events were also evaluated as secondary endpoints. Other endpoints included acceptance of the smartwatch by patients, correlation of traditional and digital 6-minute walk distance measurements, and association of digital measures with 6-minute walk distance.

### Statistical Analysis

Statistical analyses were exploratory and descriptive.

Based on a feasibility study conducted in 2018 and ongoing study experience, the final planned sample size was 25 to 50 patients. The minimum number of 25 patients was considered sufficient to obtain reasonably precise correlation coefficients (even with 40% missing data).

The safety analysis set included all patients who received at least 1 dose of inhaled iloprost. If a patient withdrew consent without agreeing to further use of their data, they were excluded from the safety analysis set. The full analysis set included all patients from the safety analysis set who had ≥3 days of activity measurements during the baseline period, ≥3 days of activity measurements during the last 2 weeks of the observation period, and data at the initial and final visits for at least 1 of the following clinical outcome measures: 6-minute walk distance, BNP/NT-proBNP levels, and WHO functional class. Patients identified as screening failures after enrollment (included by mistake) were excluded from the full analysis set.

Measurements per day were normalized, for an assumed 18 hours (6 AM to midnight) of activity per day if parts of the observational time period of that day were missing, based on the percentage of time spent wearing the smartwatch. If the watch was worn for less than 10% of a given day, data for that day were treated as missing.

Device-measured daily physical activity at baseline was calculated as the median of ≤14 daily assessments before the first intake of inhaled iloprost. Device-measured daily physical activity at the end of the observation period was calculated as the median of daily assessments in the last 14 days of the observation period.

Pearson correlations were calculated for complete, pairwise observations of traditional and digital measures included in the primary analysis. Missing data for these parameters were not imputed.

Regression analyses were performed with change from baseline in a traditional clinical measure as the dependent variable and changes from baseline in digital measures (distance walked, number of standing up events, and 6-minute walk distance) as independent variables.

## Results

### Patients

**Figure 1:**
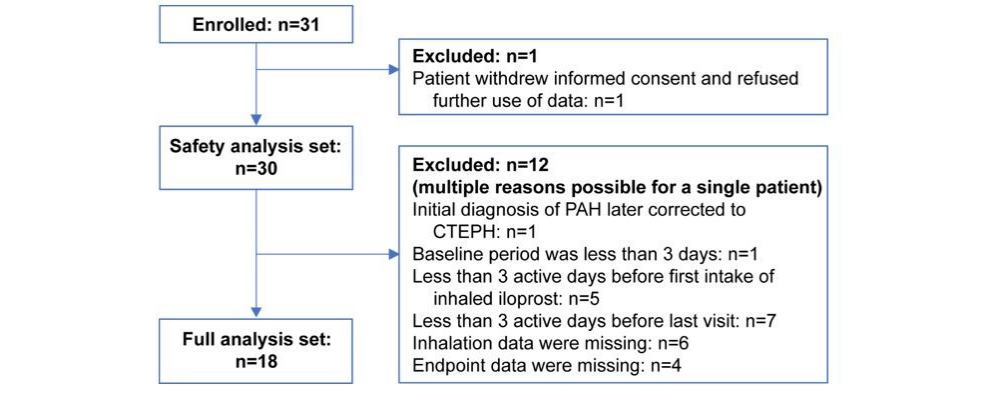
Patient disposition. CTEPH: chronic thromboembolic pulmonary hypertension; PAH: pulmonary arterial hypertension.

We screened and enrolled 31 patients; 30 patients were included in the safety analysis set, and 18 patients were included in the full analysis set (Figure 1). Most patients in the full analysis set (Multimedia Appendix 3) were older adults (aged ≥65 years; n=12), female (n=12), and White (n=17). All patients were taking oral pulmonary arterial hypertension therapies at baseline.

The most common iloprost dose at the start and end of the observation period was 2.5 μg; 3 patients changed doses (Multimedia Appendix 4). The median number of inhalation sessions per day was 5.0 (IQR 4.3 to 5.7), and the inhalation duration per session was 5.5 minutes (IQR 3.8 to 6.3). More than 75% of the inhalations were complete—the median daily percentage of complete inhalations was 100% (IQR 100% to 100%; range 50%-100%).

### Use of the Smartwatch During the Study

The median observation period in the full analysis set was 91.5 days (IQR 88.0 to 92.0; range: 34-113 days). Participants wore the smartwatch between 30 and 113 days overall (baseline period: range 4-14 days; end of the observation period: 8-14 days), for a mean time ranging from 4.8 hours per day to 11.5 hours per day (baseline period: range 4.1-14.6 hours per day; end of the observation period: range 4.7-10.5 hours per day). During the baseline period, 12 of the 18 patients (67%) wore the smartwatch for less than 7 days, and 6 of the 18 patients (33%) wore their smartwatch for at least 7 days. During the end of the observation period, almost three-quarters of the patients (13/18, 72%) wore the smartwatch every day, and all patients (18/18, 100%) wore it for at least 7 days. (Table 1). The number of hours spent wearing the watch per day decreased over the study period, but 89% of the patients (16/18) still wore the smartwatch for ≥6 hours per day on average during the end of the observation period (Table 1).

**Table 1:** Smartwatch use during the study (full analysis set).

Use characteristic			Patients, n (%)		
		Baseline period		Last 14 days of observation period	
**Number of days worn**					
	<7 days	12 (67)		0 (0)	
	≥7 days	6 (33)		18 (100)	
	≥10 days	3 (17)		17 (94)	
	14 days	2 (11)		13 (72)	
**Average daily hours worn**					
	<6 hours	2 (11)		2 (11)	
	≥6 hours	16 (89)		16 (89)	
	≥10 hours	9 (50)		4 (22)	
	≥14 hours	1 (6)		0 (0)	
	18 hours	0 (0)		0 (0)	

### Association Between Changes in Digital Physical Activity Measures and Changes in Traditional Clinical Measures

#### Comparison of Traditional and Digital Measures for 6-Minute Walk Distance

The median increase in traditional 6-minute walk distance was 26 m (Table 2). Although traditional and digital 6-minute walk distances were measured in parallel, digital 6-minute walk distance showed no substantial change from baseline (median −4.3 m, IQR −33.5 to 35.8), despite the observed change in traditionally measured 6-minute walk distance.

**Table 2:** Traditional and digital measures for 6-minute walk distance before and after 12 weeks of inhaled iloprost therapy.

Measure			Patients		Values				
			n		Median		IQR		Range
**Traditional 6-minute walk distance (meters)**									
	First visit	18		339		250 to 420		196 to 546	
	Last visit	16^a^		366		282 to 418		207 to 585	
	Change^b^	16^a^		26		0 to 40		−43 to 87	
**Digital 6-minute walk distance^c^ (meters)**									
	First visit	14		423.1		364.2 to 460.6		301.3 to 556.8	
	Last visit	14		433.9		329.2 to 486.3		267.8 to 592.6	
	Change	14		−4.3		−33.5 to 35.8		−69.2 to 90.0	

^a^Two of the 18 patients withdrew consent during the observation period and did not perform a 6-minute walk distance test at their final visit (they agreed to the use of data that had already been collected).

^b^Patients analyzed at the first and last visit were used for the calculation of change. The median change as a robust center estimate is not necessarily the difference of the median of the total distance walked.

^c^Only for patients with available values for both baseline and final visit.

#### Further Traditional Measures and Health-Related Quality of Life

Borg dyspnea index increased from baseline in 7/18 patients (39%), remained stable in 6/18 patients (33%), and decreased in 3/18 patients (17%) (Table 3). WHO functional class changed from III to II in 4 patients and remained unchanged in the other 14 patients. The EQ-5D weighted index did not change considerably.

**Table 3:** Traditional clinical measures and health-related quality of life before and after 12 weeks of inhaled iloprost therapy.

Measure			Patients		Values				
			n		Median		IQR		Range
**Borg dyspnea**									
	First visit	18		5.0		4.0 to 6.0		1.0 to 10.0	
	Last visit	16^a^		3.5		2.5 to 6.5		1.0 to 8.0	
	Change	16^a^		−0.5		−2.0 to 0.0		−3.0 to 3.0	
**EQ-5D^b^**									
	First visit	17		0.88		0.74 to 0.96		0.38 to 1.00	
	Last visit	16		0.87		0.74 to 0.97		−0.26 to 1.00	
	Change	15		0.02		0.00 to 0.08		−0.21 to 0.18	
**B-type natriuretic peptide level (ng/L)**									
	First visit	6		181		75 to 352		17 to 430	
	Last visit	6		136		44 to 166		22 to 304	
	Change	6		−16		−236 to 5		−264 to 99	
**N-terminal pro–B-type natriuretic peptide level (ng/L)**									
	First visit	10		1836		692 to 4676		78 to 15,749	
	Last visit	8		2005		759 to 3726		92 to 9859	
	Change	8		−749		−3415 to 212		−5890 to 1888	

^a^Two of the 18 patients withdrew consent during the observation period and did not perform a 6-minute walk distance test at their final visit (they agreed to the use of data that had already been collected).

^b^EQ-5D: EuroQol 5-dimension questionnaire.

#### Digital Measures of Physical Activity

Digital measures of daily physical activity (daily distance walked, number of steps per day, and number of standing-up events per day) increased from baseline to the end of the observation period (Table 4).

**Table 4:** Digital measures before and after 12 weeks of inhaled iloprost therapy.

Measure			Patients		Values				
			n		Median		IQR		Range
**Distance walked per day^a^ (km)**									
	Baseline	18		5.2		3.1 to 7.6		1.7 to 14.9	
	End of observation	18		6.5		4.0 to 7.8		2.6 to 15.7	
	Change	18		0.4		−0.2 to 1.9		−3.6 to 4.7	
**Number of steps per day^a^**									
	Baseline	18		6721		4073 to 10,258		2268 to 18,398	
	End of observation	18		8332		5330 to 10,101		3389 to 19,451	
	Change	18		591		−509 to 2413		−4605 to 6075	
**Number of standing-up events per day^a^**									
	Baseline	18		24.2		20.4 to 25.6		0.0 to 56.5	
	End of observation	18		24.8		23.1 to 26.7		14.5 to 55.0	
	Change	18		1.7		−1.1 to 5.4		−9.9 to 25.5	

^a^Average during baseline and last 14 days of observation period.

#### Correlation Between Changes From Baseline in Traditional and Digital Measures

Changes from baseline in traditional 6-minute walk distance and digital 6-minute walk distance were moderately correlated (Table 5). The numbers of patients with available data for BNP and NT-proBNP levels (n=6 and n=8, respectively) were too low for a meaningful analysis of correlation. A sensitivity analysis that had been planned (calculation using the complete data set instead of pairwise complete observations) was not performed because the volume of data was insufficient for further analysis.

**Table 5:** Pearson correlation between changes from baseline in traditional clinical parameters, health-related quality of life, and digital parameters after starting inhaled iloprost therapy.

Measure			Patients		Digital parameters, *r*						
		n		Average distance walked per day^a^		Average number of steps per day^a^		Average number of standing-up events per day^a^		6-minute walk distance^b^	
**Traditional parameters**											
	6-minute walk distance (meters)^b^	16		0.35		0.35		−0.30		0.57	
	Borg dyspnea^b^	16		−0.33		−0.33		−0.08		0.37	
	B-type natriuretic peptide level (ng/L)	6		−0.78		−0.79		0.27		−0.13	
	N-terminal pro–B-type natriuretic peptide level (ng/L)	8		−0.01		−0.02		0.11		−0.29	
EQ-5D^c^ weighted index			15		0.14		0.14		0.05		0.52

^a^Calculation based on a daily period from 6 AM to midnight.

^b^Two of the 18 patients withdrew consent during the observation period and did not perform a 6-minute walk distance test at their final visit (they agreed to the use of data that had already been collected).

^c^EQ-5D: EuroQol 5-dimension questionnaire.

### Heart Rate

The median average heart rate at rest was 75.9 beats per minute (IQR 70.4 to 88.5) at baseline and 76.5 beats per minute (IQR 69.5 to 85.4) at the final visit. From baseline to the final visit, the average heart rate at rest showed a median decrease of −1.3 beats per minute (IQR −8.0 to 3.5).

**Figure 2:**
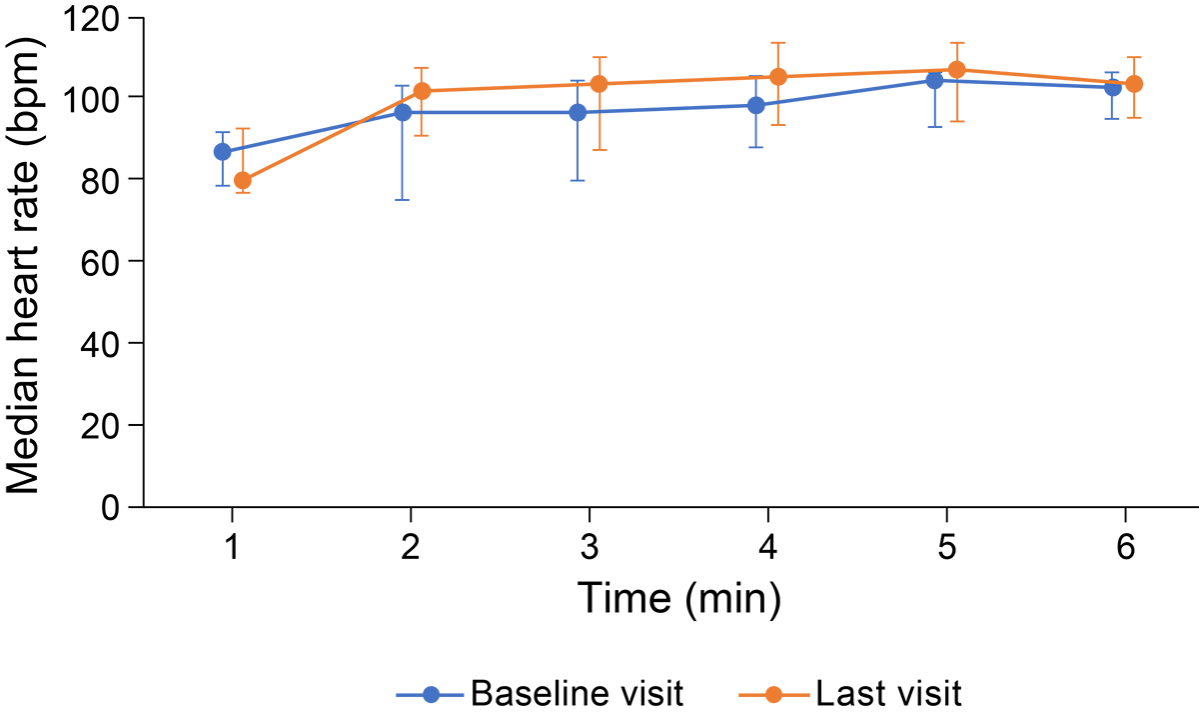
Median heart rate Data collected by smartwatch during 6-minute walk distance test before (baseline) and under (last visit) inhaled iloprost therapy. Error bars show IQR.

**Figure 3:**
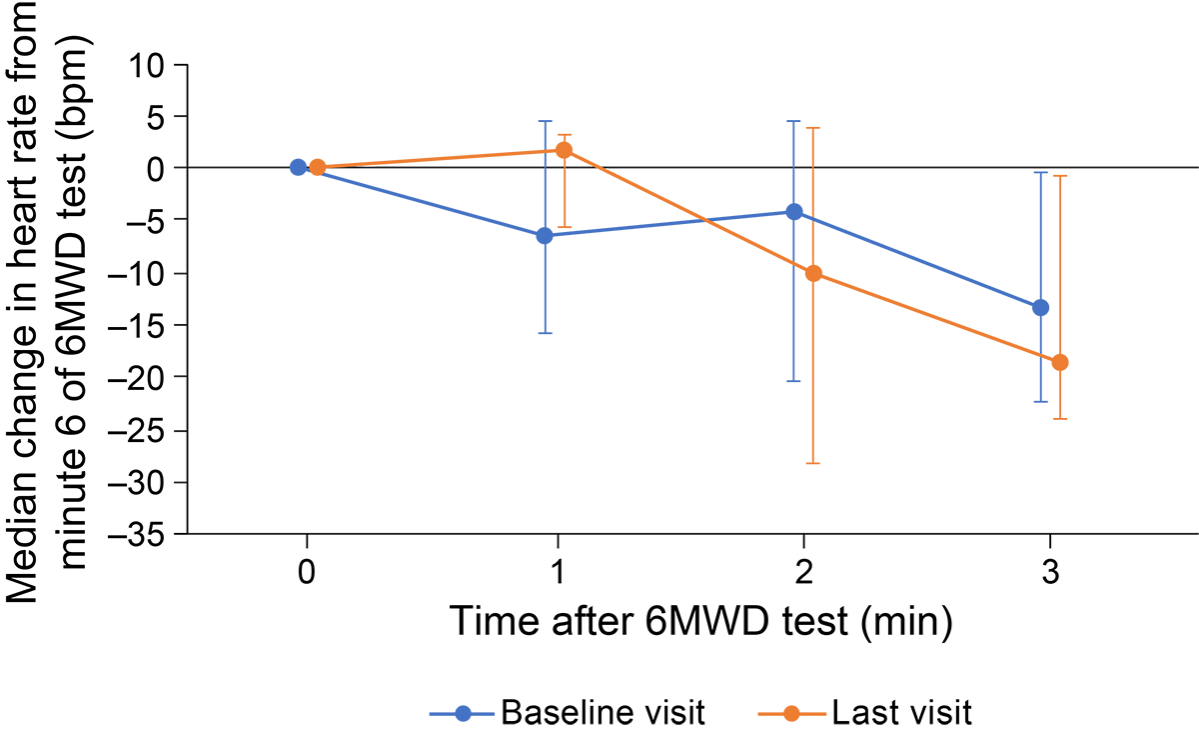
Heart rate recovery data collected by smartwatch at the end of the 6-minute walk distance (6MWD) test before (baseline) and under (last visit) inhaled iloprost therapy. Error bars show IQR.

Heart rates measured during the 6-minute walk distance test were slightly higher at the last visit compared with those at the baseline visit (Figure 2). The median changes in heart rate from 1 minute before the 6-minute walk distance test to each minute during the 6-minute walk distance test were also higher at the last visit (4.6 beats per minute to 23.9 beats per minute) than those at the baseline visit (3.0 beats per minute to 20.5 beats per minute) (Multimedia Appendix 5). Heart rate recovery immediately after the 6-minute walk distance test was slower at the last visit than that at the baseline visit (Figure 3).

### Inhalation and Changes in Heart Rate and Physical Activity

The study design enabled parallel digital tracking of iloprost inhalation, heart rate, and physical activity. An analysis of patients stratified by the median duration of iloprost inhalation and treatment month suggested that the average heart rate during iloprost inhalation showed a slight increase with duration of inhalation (Multimedia Appendix 6). Owing to large differences in subgroup size, no clear trend for the change in average heart rate during treatment could be detected.

**Figure 4:**
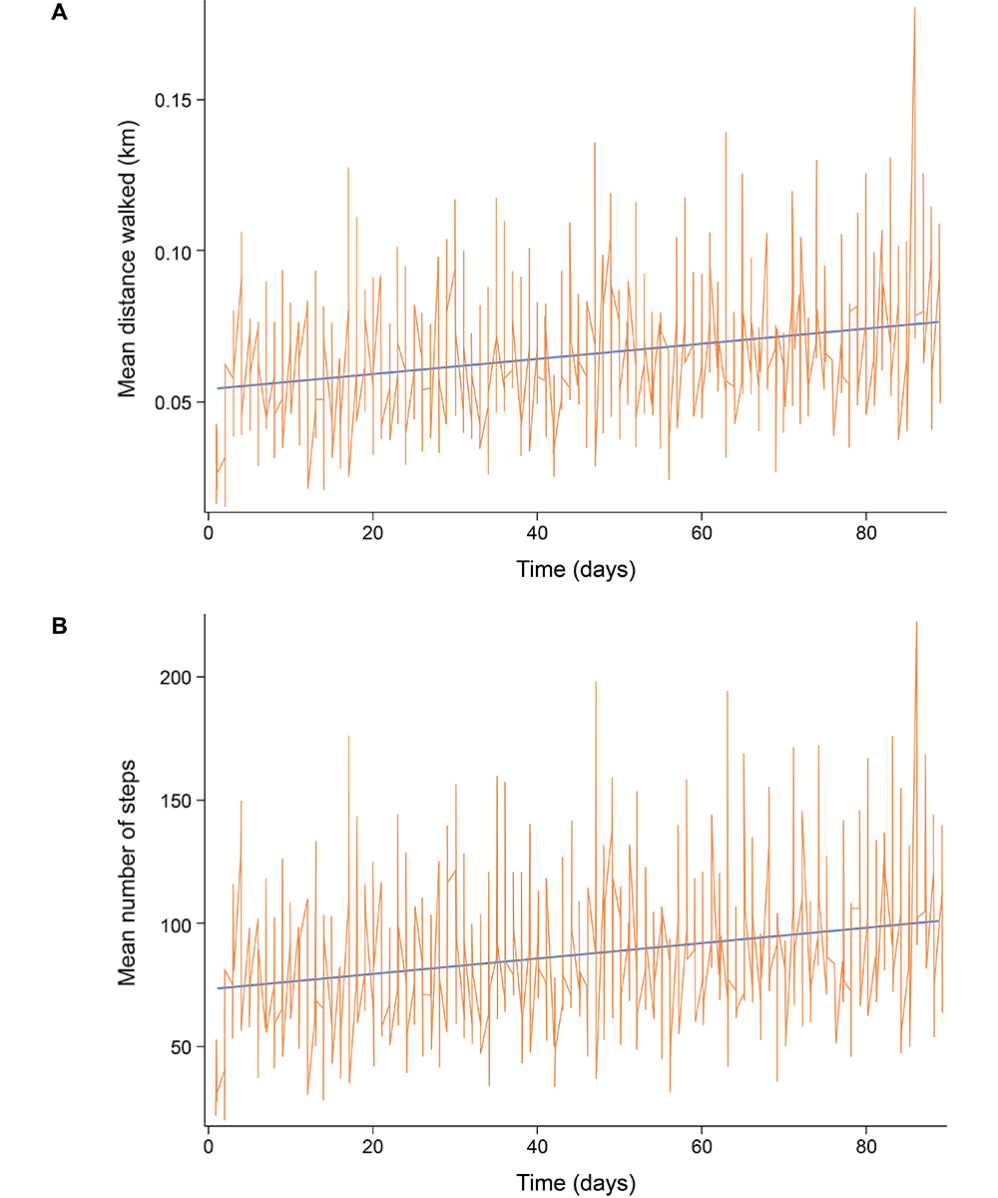
(A) Distance walked and (B) number of steps (measured at 15-minute intervals for 3 hours after the end of each iloprost inhalation session) over the whole observation period.

Distance walked (measured digitally) showed a short-term increase in activity shortly after inhalation and a long-term increase in activity over the entire observation period (Figure 4A). A similar association was observed for the number of steps (Figure 4B) but was not observed for the number of standing-up events (not shown).

For each iloprost inhalation session per day, 12 intervals (of 15 minutes) beginning at the end of the inhalation session were defined, and the distance walked per interval was recorded. The corresponding intervals for each inhalation per day were averaged for each patient (ie, the first intervals after the inhalations were averaged, then the second intervals after the inhalations, etc) The resulting 12 average values for each patient each day were then averaged over all patients. Complete and incomplete inhalations were included. Approximately 3700 inhalations were excluded from the analysis because the watch was not worn over the entire period of 3 hours after the end of inhalation. Some of the 3-hour intervals were overlapping. The coverage for the first analyzed 15-minute interval of each inhalation could be less than 100%, which occurred about 40 times. Within 1 day, the intervals after the inhalations were not distributed equally over the day.

### Additional Analyses

In regression analyses, the change from baseline in traditional 6-minute walk distance was associated with changes in digital outcomes (distance walked, number of standing-up events, and digital 6-minute walk distance). Absolute measurements of traditional and digital 6-minute walk distance were strongly correlated but not congruent at baseline (*r*=0.86) and at the final visit (*r*=0.87). Further details of these and other additional analyses are presented in Multimedia Appendix 7.

## Discussion

### Feasibility of Digital Monitoring

To the best of our knowledge, this study is the first to monitor physical activity and heart rate using iPhones and Apple Watches in patients with pulmonary arterial hypertension. The results demonstrate the feasibility of using digital parameters as digital biomarkers for long-term activity levels in patients with pulmonary arterial hypertension. Despite the fact that patients were blinded to smartwatch-collected data, acceptance was good—almost 90% wore the smartwatch for at least 6 hours per day at baseline (16/18, 89%) and for the end of the observation period (16/18, 89%). Although correlations between changes in digital and traditional clinical outcome measures were few, the overall directions of those changes were generally concordant. The results also demonstrated the feasibility of continuously measuring multiple digital parameters (eg, inhalations, heart rate, and physical activity) in parallel with different devices and matching the data. We were able to detect short-term increases in physical activity after each iloprost inhalation session with fine granularity. The results also highlighted some important challenges and limitations that should be considered for future digital studies in patients with pulmonary arterial hypertension.

Recruitment was limited by a lack of available patients, which resulted in a small study population. This could be partly due to the rarity of pulmonary arterial hypertension [17], the willingness of patients to undergo digital monitoring, and the suitability of the study devices for the patients [14]. Nevertheless, data were fully available in the full analysis set for most outcome variables. Thus, reasonable precision was obtained for most correlation coefficients in the primary endpoint analysis, with the exceptions of BNP and NT-proBNP levels; as no central laboratory facility was used, clinicians generally measured either BNP or NT-proBNP (rather than both) based on local availability, which meant that few patients had data for each individual laboratory parameter.

### Comparison Between Clinical and Digital Parameters

Although there was general agreement between changes in digital and traditional parameters from baseline, parallel digital and traditional measurements of 6-minute walk distance were not congruent. The proprietary digital 6-minute walk distance algorithm was based on step count and the step length of healthy individuals, which in general is not valid for patients with pulmonary arterial hypertension. Substitution of the healthy step length with a patient’s individualized step length could provide better estimation of distance. The current series of Apple devices includes individual and repeated measurement of step length, which may be used in future studies to better estimate distance. This feature may also enable researchers to characterize other changes in health status (eg, dyspnea) more precisely. Alternatively, measurement of distance by GPS may be suitable if the 6-minute walk distance test can be performed outdoors. Wi-Fi could be a potential alternative to GPS for 6-minute walk distance tests conducted indoors. However, measurement of 6-minute walk distance by GPS or Wi-Fi would require precise location tracking. A feasibility survey suggested that precise tracking on a long-term basis would not be accepted by patients [14]; such tracking would, therefore, need to be restricted to during the 6-minute walk distance test (eg, by designing the 6-minute walk distance app to automatically switch GPS and Wi-Fi tracking on at the start of the 6-minute walk distance test and off at the end of the test).

Previous digital studies [18-22] with patients with pulmonary arterial hypertension have shown mean step counts in the range of 3234 to 5041 and mean traditional 6-minute walk distances in the range of 343 m to 458 m. In comparison, this study population had higher step counts (median 6721 at baseline and 8332 at the end of the observation period) but broadly similar traditional 6-minute walk distance (median 339 m at baseline and 366 m at the final visit). The reasons for this are unclear but may include methodological differences and the small sample size in our study. Patients in previous studies were monitored for only short periods of time (≥3 days [18], ≥3 weekdays [20], 6 days on average [21,22], or 7 days [19]), whereas patients in our study were monitored for 30 to 113 days (including 4-14 days in the baseline period and 8-14 days at the end of the observation period). Studies [23,24] with healthy adults have shown variations in physical activity over the course of a week, with the lowest levels of inactivity occurring on Saturdays. The results in patients with pulmonary arterial hypertension for shorter duration study periods may, therefore, have been influenced by the timing of physical activity monitoring. Mainguy et al [19] evaluated patients for 7 consecutive days but asked them to avoid performing unfamiliar activities during that time; this restriction was not used in our study.

The number of standing-up events is difficult to interpret. In a recent study with patients with pulmonary arterial hypertension, sit-to-stand test results (the number of sit-to-stand movements that patients were able to complete in 30 seconds) were associated with quadriceps muscle strength, which in turn was associated with 6-minute walk distance [25]. An official statement from the European Respiratory Society [26] noted that the sit-to-stand test depends substantially more on muscle strength, equilibrium, and gait balance than on the mechanisms of oxygen and carbon dioxide transport that are the target of vasoactive pulmonary arterial hypertension treatments. During long-term monitoring, an increase in the number of standing-up events per day could reflect an increase in distance walked or an increase in sitting-down events (in the absence of an increase in activity, the latter would suggest a worsening of the patient’s condition). The assessment of standing-up events in patients with pulmonary arterial hypertension is not yet well established, and more data are needed.

The physical activity level may also be influenced by factors other than the severity of pulmonary arterial hypertension (eg, patient preferences, patient behavior, or the season in which the patient was recruited). Nevertheless, daily activity has been linked to prognosis in patients with pulmonary hypertension [27].

The abovementioned limitations provide examples of some of the many criteria that must be fulfilled to ensure a successful digital study. The criteria can be broadly categorized into 6 themes, collectively referred to as the digital real-world evidence matrix: patient (eg, patient willingness to use digital devices and preferences regarding design and features of digital devices), indication (eg, number of available patients and suitability of disease-relevant outcome measures for digital monitoring), biomarker (eg, connection of digital measure to a health-related outcome), type of sensor or device (eg, ease of use, personalization, and data security), quality (eg, accuracy of primary data capture), and endpoint (eg, transformation of sensor data into a meaningful, patient-centered endpoint). These themes were addressed in the design of the VENTASTEP study, which combined digital and traditional monitoring to provide new insights into the real-life treatment of patients with pulmonary arterial hypertension.

### Changes in Digital and Traditional Measures After Initiation of Inhaled Iloprost

There was an increase of 26 m for traditional investigator-measured 6-minute walk distance from baseline after addition of inhaled iloprost to oral pulmonary arterial hypertension therapy, which is consistent with the results of previous clinical trials [10,11]. The increase in traditional 6-minute walk distance was accompanied by changes in other traditional parameters and digital measures of daily physical activity.

6-Minute walk distance assessed during an office visit may be affected by daily fluctuations in physical function and may not accurately capture long-term changes in symptoms and physical function. Furthermore, the 6-minute walk distance test reflects maximal activity, whereas daily physical activity parameters provide an average of overall activity. Digital monitoring provides new insights into patient activity, with a more complete picture of the patient’s health status than traditional 6-minute walk distance alone. For example, the digital data showed that physical activity increased shortly after each iloprost inhalation session. This sheds light on the day-to-day experiences of patients with pulmonary arterial hypertension taking inhaled iloprost and may be helpful information for patients or physicians considering iloprost therapy.

The increase in heart rate during the 6-minute walk distance test was marginally greater at the final visit compared with that the baseline visit, and heart rate recovery time after the 6-minute walk distance test increased. A previous study [28] showed significant associations between the chronotropic response (peak walking heart rate minus resting heart rate) and 6-minute walk distance in patients with pulmonary arterial hypertension, which led Provencher et al [28] to suggest that a lack of chronotropic response may reflect a loss of normal physiological reserve in patients who were more unwell. Preservation of the chronotropic response may therefore be a positive sign. However, given the high variability of heart rate in our study, changes to this parameter cannot be clearly interpreted.

Despite increases in daily physical activity over the course of the study, the EQ-5D weighted index showed little change. The EQ-5D score has produced mixed results in key clinical trials of pulmonary arterial hypertension therapies [9], showing improvements in placebo-controlled trials of sildenafil [29] and tadalafil [30] but not in a placebo-controlled trial of riociguat [31], despite riociguat resulting in patients showing significant improvements in traditional measures such as 6-minute walk distance and WHO functional class. The EQ-5D is a generic instrument [9] and may therefore lack specificity to address the important health-related quality of life issues in pulmonary arterial hypertension [8]. Health-related quality of life may also be influenced by associated comorbidities such as scleroderma, liver cirrhosis, and HIV [32].

Sleep disturbance in patients with pulmonary arterial hypertension has been associated with symptom severity, psychological state, and health-related quality of life [33]. PSQI score showed slight changes in sleep quality from baseline to the final visit. A global PSQI score of 5 has been proposed as a cut-off to distinguish good and poor sleep [34]. Thus, sleep quality in the study group as a whole shifted from poor to good during the course of the study.

### Strengths and Limitations

The strengths of this study include its continuous, parallel, digital measurement of inhalation behavior, heart rate, and physical activity over a long observation period. The limitations of this study include its small sample size, insufficient BNP or NT-proBNP data, and the fact that the digital 6-minute walk distance algorithm was based on the step length of healthy individuals rather than that of patients with pulmonary arterial hypertension. A substantial proportion of the study population took concomitant beta-blockers, which should be considered when interpreting the heart rate data.

### Implications for Future Research

Further research is warranted to assess the association of iloprost inhalation behavior with changes in digital physical activity measures, traditional clinical measures, and health-related quality of life. The relationship between daily physical activity and traditional 6-minute walk distance requires further exploration. In addition, studies with longer-term follow-up are needed to determine if clinical outcomes (eg, mortality or time to clinical worsening) are better predicted by the change in traditional 6-minute walk distance or the change in digital 6-minute walk distance over time.

The recruitment difficulties in this study (which focused on a rare disease) suggest that digital monitoring studies may be more feasible in indications with large patient populations, such as heart failure. In addition, most patients were older adults; recruitment may be higher among populations of younger patients who are more familiar with digital technology. Refinement of the digital 6-minute walk distance algorithm for the target patient population is also needed.

### Conclusions

Although generalizability is limited because of the small sample size and other limitations, the study demonstrated the general feasibility of performing digital assessments with a commercially available smartwatch and smartphone in patients with pulmonary arterial hypertension. Digital measures of daily physical activity and traditional clinical measures showed concordant changes from baseline after addition of inhaled iloprost to oral pulmonary arterial hypertension therapies. However, the data indicate that activity tracking algorithms validated for healthy people require adaptation for patients with pulmonary arterial hypertension. Further investigations are therefore necessary.

## Appendix

Multimedia Appendix 1 Amendment to protocol.

Multimedia Appendix 2 Additional information on digital parameters.

Multimedia Appendix 3 Patient characteristics at baseline.

Multimedia Appendix 4 Iloprost dose and inhalation characteristics.

Multimedia Appendix 5 Change in heart rate from 1 minute before the 6MWD test to each minute during the 6MWD test (full analysis set).

Multimedia Appendix 6 Average heart rate during iloprost inhalation per month of observation, stratified by median length of inhalation (full analysis set).

Multimedia Appendix 7 Additional results – secondary objectives.

## Abbreviations

BNP: B-type natriuretic peptide
EQ-5D: EuroQol 5-dimension questionnaire
NT-proBNP: N-terminal pro–B-type natriuretic peptide
PSQI: Pittsburgh Sleep Quality Index
WHO: World Health Organization
