# Supplementary material for: Digital Tracking of Physical Activity, Heart Rate, and Inhalation Behavior in Patients With Pulmonary Arterial Hypertension Treated With Inhaled Iloprost: Observational Study (VENTASTEP)
Source: J Med Internet Res. 2021 Oct 8;23(10):e25163. doi: 10.2196/25163 (PMC8538027; doi:10.2196/25163)
Supplement: Multimedia Appendix 1 [file jmir_v23i10e25163_app1.doc]

## Multimedia Appendix 1

**Digital Tracking of Physical Activity, Heart Rate, and Inhalation Behavior in Patients With Pulmonary Arterial Hypertension treated With Inhaled Iloprost: Observational Study (VENTASTEP)**

Barbara Stollfuss^1^, MD, PhD; Manuel Richter^2^, MD; Daniel Drömann^3^, MD; Hans Klose^4^, MD; Martin Schwaiblmair^5^, MD; Ekkehard Grünig^6^, MD; Ralf Ewert^7^, MD; Martin C Kirchner^1^, Dipl-Biol; Frank Kleinjung^8^, PhD; Valeska Irrgang^1^, MD; Christian Mueller^1^, PhD

**Table.** Amendment to protocol.

| **Amendment effective date** | **Reasons for amendment** |
| --- | --- |
| 8 January 2019 | Reduction of target sample size to 25 at a minimum and 50 at a maximum |
|  | Prolongation of recruitment period until Q3 2019 |
|  | New patient support program supplier by 1 January 2019 (Contra Care GmbH was replaced by Vitartis Medizin Service GmbH) |
