# Supplementary material for: Digital Tracking of Physical Activity, Heart Rate, and Inhalation Behavior in Patients With Pulmonary Arterial Hypertension Treated With Inhaled Iloprost: Observational Study (VENTASTEP)
Source: J Med Internet Res. 2021 Oct 8;23(10):e25163. doi: 10.2196/25163 (PMC8538027; doi:10.2196/25163)
Supplement: Multimedia Appendix 2 [file jmir_v23i10e25163_app2.doc]

## Multimedia Appendix

**Digital Tracking of Physical Activity, Heart Rate, and Inhalation Behavior in Patients With Pulmonary Arterial Hypertension treated With Inhaled Iloprost: Observational Study (VENTASTEP)**

Barbara Stollfuss^1^, MD, PhD; Manuel Richter^2^, MD; Daniel Drömann^3^, MD; Hans Klose^4^, MD; Martin Schwaiblmair^5^, MD; Ekkehard Grünig^6^, MD; Ralf Ewert^7^, MD; Martin C Kirchner^1^, Dipl-Biol; Frank Kleinjung^8^, PhD; Valeska Irrgang^1^, MD; Christian Mueller^1^, PhD

## Additional Information on Digital Parameters

Distance walked was estimated through the pedometer (based on step count and a stride length algorithm trained on healthy volunteer data) and GPS. The number of steps was estimated through the dedicated motion coprocessor of the smartphone, using accelerometer and gyroscope data from the smartwatch. The number of standing-up events was calculated based on wrist position. Digital 6-minute walk distance (6MWD) was based on step count and the aforementioned stride length algorithm (as the 6MWD test is typically performed indoors, the ability to measure distance walked by GPS was limited). Other planned digital parameters (number of floors climbed, time spent at home, number of relevant location changes, and number of times leaving home [14]) were not included in the final analysis because they could only be measured by the smartphones (not the smartwatches), and it was not possible to ascertain whether the patients always carried the smartphones with them.
