# Supplementary material for: Digital Tracking of Physical Activity, Heart Rate, and Inhalation Behavior in Patients With Pulmonary Arterial Hypertension Treated With Inhaled Iloprost: Observational Study (VENTASTEP)
Source: J Med Internet Res. 2021 Oct 8;23(10):e25163. doi: 10.2196/25163 (PMC8538027; doi:10.2196/25163)
Supplement: Multimedia Appendix 3 [file jmir_v23i10e25163_app3.doc]

## Multimedia Appendix 3

**Digital Tracking of Physical Activity, Heart Rate, and Inhalation Behavior in Patients With Pulmonary Arterial Hypertension treated With Inhaled Iloprost: Observational Study (VENTASTEP)**

Barbara Stollfuss^1^, MD, PhD; Manuel Richter^2^, MD; Daniel Drömann^3^, MD; Hans Klose^4^, MD; Martin Schwaiblmair^5^, MD; Ekkehard Grünig^6^, MD; Ralf Ewert^7^, MD; Martin C Kirchner^1^, Dipl-Biol; Frank Kleinjung^8^, PhD; Valeska Irrgang^1^, MD; Christian Mueller^1^, PhD

**Table.** Patient characteristics at baseline.

|  |  | | **Patients with PAH (N=18)** |
| --- | --- | --- | --- |
|  |  | |  |
| **Age at inclusion, n (%)** | | |  |
|  | <65 years | | 6 (33) |
|  | ≥65 years | | 12 (67) |
| **Sex, n (%)** | | |  |
|  | Male | | 6 (33) |
|  | Female | | 12 (67) |
| **Ethnic origin, n (%)^a^** | | |  |
|  | White | | 17 (94) |
|  | Black or African American | | 1 (6) |
|  | Asian | | 0 |
|  | Not reported | | 1 (6) |
| Median [IQR] BMI, kg/m^2^ | | | 25.5 [22.5, 28.7] |
| Median [IQR] systolic blood pressure, mmHg | | | 110 [100, 120] |
| Median [IQR] diastolic blood pressure, mmHg | | | 69 [59, 73] |
| Median [IQR] heart rate, bpm | | | 74 [67, 83] |
| **Education level, n (%)** | | |  |
|  | Elementary education | | 4 (22) |
|  | General certificate for secondary education | | 6 (33) |
|  | University entrance qualification | | 1 (6) |
|  | Not reported | | 7 (39) |
| **Smoking status, n (%)** | | |  |
|  | Never | | 8 (44) |
|  | Former | | 9 (50) |
|  | Current | | 1 (6) |
| **Most common comorbidities, n (%)** | | |  |
|  | Hypertension | | 12 (67) |
|  | Chronic obstructive pulmonary disease | | 5 (28) |
|  | Coronary artery disease | | 5 (28) |
|  | Hyperlipidemia | | 5 (28) |
| Median [IQR] duration since PAH diagnosis, months | | | 18.9 [5.3, 69.9] |
| **Pre-existing PAH therapy at baseline, n (%)** | | |  |
|  | Monotherapy | | 8 (44) |
|  |  | sGC stimulator | 2 (11) |
|  |  | PDE5 inhibitor | 2 (11) |
|  |  | ERA | 4 (22) |
|  | Dual combination therapy | | 10 (56) |
|  |  | ERA+sGC stimulator | 4 (22) |
|  |  | ERA+PDE5 inhibitor | 6 (33) |

^a^Multiple responses were possible.

bpm: beats per minute; ERA: endothelin receptor antagonist; PAH: pulmonary arterial hypertension; PDE5: phosphodiesterase type 5; sGC: soluble guanylate cyclase.
