# Supplementary material for: Digital Tracking of Physical Activity, Heart Rate, and Inhalation Behavior in Patients With Pulmonary Arterial Hypertension Treated With Inhaled Iloprost: Observational Study (VENTASTEP)
Source: J Med Internet Res. 2021 Oct 8;23(10):e25163. doi: 10.2196/25163 (PMC8538027; doi:10.2196/25163)
Supplement: Multimedia Appendix 4 [file jmir_v23i10e25163_app4.doc]

## Multimedia Appendix 4

**Digital Tracking of Physical Activity, Heart Rate, and Inhalation Behavior in Patients With Pulmonary Arterial Hypertension treated With Inhaled Iloprost: Observational Study (VENTASTEP)**

Barbara Stollfuss^1^, MD, PhD; Manuel Richter^2^, MD; Daniel Drömann^3^, MD; Hans Klose^4^, MD; Martin Schwaiblmair^5^, MD; Ekkehard Grünig^6^, MD; Ralf Ewert^7^, MD; Martin C Kirchner^1^, Dipl-Biol; Frank Kleinjung^8^, PhD; Valeska Irrgang^1^, MD; Christian Mueller^1^, PhD

**Table.** Iloprost dose and inhalation characteristics.

|  |  | **Patients with PAH (N=18)** |
| --- | --- | --- |
|  |  |  |
| Inhaled iloprost dose, n (%) | |  |
|  | 2.5 μg throughout | 12 (67) |
|  | 5.0 μg throughout | 3 (17) |
|  | 2.5 μg → 5.0 μg^a^ | 2 (11) |
|  | 2.5 μg → 5.0 μg^a^ → 2.5 μg^b^ | 1 (6) |

^a^Change to maintenance dose. ^b^5.0 μg dose not tolerated.

PAH: pulmonary arterial hypertension.
