# Supplementary material for: Digital Tracking of Physical Activity, Heart Rate, and Inhalation Behavior in Patients With Pulmonary Arterial Hypertension Treated With Inhaled Iloprost: Observational Study (VENTASTEP)
Source: J Med Internet Res. 2021 Oct 8;23(10):e25163. doi: 10.2196/25163 (PMC8538027; doi:10.2196/25163)
Supplement: Multimedia Appendix 5 [file jmir_v23i10e25163_app5.doc]

## Multimedia Appendix 5

**Digital Tracking of Physical Activity, Heart Rate, and Inhalation Behavior in Patients With Pulmonary Arterial Hypertension treated With Inhaled Iloprost: Observational Study (VENTASTEP)**

Barbara Stollfuss^1^, MD, PhD; Manuel Richter^2^, MD; Daniel Drömann^3^, MD; Hans Klose^4^, MD; Martin Schwaiblmair^5^, MD; Ekkehard Grünig^6^, MD; Ralf Ewert^7^, MD; Martin C Kirchner^1^, Dipl-Biol; Frank Kleinjung^8^, PhD; Valeska Irrgang^1^, MD; Christian Mueller^1^, PhD

**Table.** Change in heart rate from 1 minute before the 6MWD test to each minute during the 6MWD test (full analysis set).

|  |  | **Change in heart rate, bpm** | | |
| --- | --- | --- | --- | --- |
|  | **Non-missing** | **Median** | **[IQR]** | **(Range)** |
| **At baseline visit** |  |  |  |  |
| Change from 1 minute before 6MWD to minute 1 during 6MWD | 15 | 3.0 | [−2.0, 7.4] | (−12.0, 15.8) |
| Change from 1 minute before 6MWD to minute 2 during 6MWD | 14 | 11.3 | [−2.0, 23.8] | (−26.5, 66.0) |
| Change from 1 minute before 6MWD to minute 3 during 6MWD | 13 | 17.8 | [3.5, 24.2] | (−31.5, 70.5) |
| Change from 1 minute before 6MWD to minute 4 during 6MWD | 14 | 17.4 | [11.7, 24.0] | (−13.2, 52.0) |
| Change from 1 minute before 6MWD to minute 5 during 6MWD | 15 | 20.5 | [11.4, 25.0] | (−10.7, 41.7) |
| Change from 1 minute before 6MWD to minute 6 during 6MWD | 15 | 14.6 | [11.6, 33.0] | (−17.0, 39.3) |
| **At final visit** |  |  |  |  |
| Change from 1 minute before 6MWD to minute 1 during 6MWD | 12 | 4.6 | [2.9, 7.0] | (−1.6, 10.7) |
| Change from 1 minute before 6MWD to minute 2 during 6MWD | 12 | 18.6 | [12.1, 34.3] | (−2.8, 58.2) |
| Change from 1 minute before 6MWD to minute 3 during 6MWD | 12 | 20.4 | [13.4, 35.1] | (4.9, 51.0) |
| Change from 1 minute before 6MWD to minute 4 during 6MWD | 12 | 22.2 | [19.4, 38.7] | (15.7, 48.9) |
| Change from 1 minute before 6MWD to minute 5 during 6MWD | 12 | 22.4 | [16.8, 40.6] | (12.0, 49.4) |
| Change from 1 minute before 6MWD to minute 6 during 6MWD | 12 | 23.9 | [18.1, 34.1] | (−2.1, 49.6) |

6MWD: 6-minute walk distance; bpm: beats per minute; IQR: interquartile range.
