# Supplementary material for: Digital Tracking of Physical Activity, Heart Rate, and Inhalation Behavior in Patients With Pulmonary Arterial Hypertension Treated With Inhaled Iloprost: Observational Study (VENTASTEP)
Source: J Med Internet Res. 2021 Oct 8;23(10):e25163. doi: 10.2196/25163 (PMC8538027; doi:10.2196/25163)
Supplement: Multimedia Appendix 6 [file jmir_v23i10e25163_app6.doc]

## Multimedia Appendix 6

**Digital Tracking of Physical Activity, Heart Rate, and Inhalation Behavior in Patients With Pulmonary Arterial Hypertension treated With Inhaled Iloprost: Observational Study (VENTASTEP)**

Barbara Stollfuss^1^, MD, PhD; Manuel Richter^2^, MD; Daniel Drömann^3^, MD; Hans Klose^4^, MD; Martin Schwaiblmair^5^, MD; Ekkehard Grünig^6^, MD; Ralf Ewert^7^, MD; Martin C Kirchner^1^, Dipl-Biol; Frank Kleinjung^8^, PhD; Valeska Irrgang^1^, MD; Christian Mueller^1^, PhD

**Table.** Average heart rate during iloprost inhalation per month of observation, stratified by median length of inhalation (full analysis set).

|  |  | **Heart rate during inhalation, bpm** | | |
| --- | --- | --- | --- | --- |
| **Median length of inhalation** | **Non-missing** | **Median** | **[IQR]** | **(Range)** |
| Month 1: | | | |  |
| <3 minutes | 102 | 73.8 | [70.7, 77.1] | (62.2, 106.4) |
| 3–<5 minutes | 450 | 79.2 | [74.7, 84.6] | (42.8, 197.3) |
| 5–<8 minutes | 548 | 77.1 | [72.4, 82.2] | (36.3, 198.9) |
| Month 2: | | | |  |
| <3 minutes | 156 | 73.1 | [69.9, 77.4] | (63.0, 136.6) |
| 3–<5 minutes | 601 | 78.4 | [72.4, 84.5] | (60.2, 188.6) |
| 5–<8 minutes | 745 | 75.6 | [70.3, 82.9] | (35.7, 202.0) |
| 8–<13 minutes | 81 | 81.6 | [76.2, 86.6] | (65.2, 98.1) |
| Month 3: | | | |  |
| <3 minutes | 54 | 71.6 | [68.6, 74.7] | (63.0, 85.9) |
| 3–<5 minutes | 543 | 80.5 | [74.2, 89.5] | (59.3, 203.0) |
| 5–<8 minutes | 543 | 75.7 | [69.3, 83.8] | (35.0, 194.0) |
| 8–<13 minutes | 260 | 79.8 | [74.6, 84.5] | (62.8, 104.5) |
| Month 4: | | | |  |
| 3–<5 minutes | 36 | 87.8 | [79.4, 91.2] | (29.0, 152.7) |
| 5–<8 minutes | 74 | 87.6 | [82.3, 90.9] | (60.0, 186.9) |
| 8–<13 minutes | 116 | 78.3 | [73.9, 82.8] | (63.8, 101.4) |

bpm: beats per minute; IQR: interquartile range.
