# Supplementary material for: Digital Tracking of Physical Activity, Heart Rate, and Inhalation Behavior in Patients With Pulmonary Arterial Hypertension Treated With Inhaled Iloprost: Observational Study (VENTASTEP)
Source: J Med Internet Res. 2021 Oct 8;23(10):e25163. doi: 10.2196/25163 (PMC8538027; doi:10.2196/25163)
Supplement: Multimedia Appendix 7 [file jmir_v23i10e25163_app7.doc]

## Multimedia Appendix 7

**Digital Tracking of Physical Activity, Heart Rate, and Inhalation Behavior in Patients With Pulmonary Arterial Hypertension treated With Inhaled Iloprost: Observational Study (VENTASTEP)**

Barbara Stollfuss^1^, MD, PhD; Manuel Richter^2^, MD; Daniel Drömann^3^, MD; Hans Klose^4^, MD; Martin Schwaiblmair^5^, MD; Ekkehard Grünig^6^, MD; Ralf Ewert^7^, MD; Martin C Kirchner^1^, Dipl-Biol; Frank Kleinjung^8^, PhD; Valeska Irrgang^1^, MD; Christian Mueller^1^, PhD

## Additional Results – Secondary Objectives

#### Regression Analysis of Changes From Baseline in Traditional Measures, Health-Related Quality of Life, and Digital Measures

The change from baseline in traditional 6-minute walk distance (6MWD) was associated with the changes from baseline in digital outcomes (*P*=.027; parameter estimates: 5.71, −1.31, and 0.41 for distance walked, number of standing-up events, and digital 6MWD, respectively), whereas the changes from baseline of the EuroQol five-dimensions weighted index (*P*=.377; parameter estimates: 0.0046, 0.0003, and 0.0011) and N-terminal pro-brain natriuretic peptide (*P*=.706; parameter estimates: 559.38, −9.30, and −3.56) were not. Owing to limited data, regression analyses of other traditional measures were not performed.

#### Correlations of Absolute Measurements

The correlation between traditional 6MWD and digital 6MWD was strongly positive at baseline (*r*=0.86) and remained so at the final visit (*r*=0.87), despite the change from baseline being higher for traditional 6MWD than digital 6MWD (mean differences do not affect correlations).

The correlation between average heart rate during 6MWD and average number of steps per day (14 days before first intake or last visit) was not significant (baseline: *r*=0.31; last visit: *r*=0.29).

#### Sleep Quality

Sleep quality was only slightly impaired at the initial visit (median Pittsburgh Sleep Quality Index [PSQI] subscale scores between 0 and 1), but still showed improvement during the study (see Table below). The median [IQR] total PSQI score decreased from 6.0 [3.0, 7.0] at baseline to 4.0 [3.0, 6.0] after treatment at the final visit.

**Table.** Change in PSQI from baseline visit to final visit (full analysis set).

| **PSQI subscale** | **Non-missing** | **Median** | **[IQR]** | **(Range)** |
| --- | --- | --- | --- | --- |
| Sleep duration | | | |  |
| Initial visit | 18 | 0.0 | [0.0, 1.0] | (0.0, 2.0) |
| Final visit | 16 | 0.0 | [0.0, 0.5] | (0.0, 2.0) |
| Change | 16 | 0.0 | [0.0, 0.0] | (−1.0, 0.0) |
| Sleep disturbance | | | |  |
| Initial visit | 16 | 1.0 | [1.0, 1.5] | (0.0, 2.0) |
| Final visit | 15 | 1.0 | [1.0, 1.0] | (1.0, 2.0) |
| Change | 13 | 0.0 | [0.0, 0.0] | (−1.0, +1.0) |
| Sleep latency | | | |  |
| Initial visit | 16 | 1.0 | [0.0, 2.0] | (0.0, 3.0) |
| Final visit | 16 | 1.0 | [0.0, 1.0] | (0.0, 3.0) |
| Change | 14 | 0.0 | [−1.0, 0.0] | (−2.0, +1.0) |
| Day dysfunction due to sleepiness | | | |  |
| Initial visit | 18 | 1.0 | [1.0, 2.0] | (0.0, 3.0) |
| Final visit | 16 | 1.0 | [1.0, 1.0] | (0.0, 2.0) |
| Change | 16 | 0.0 | [−1.0, 0.0] | (−2.0, +1.0) |
| Sleep efficiency | | | |  |
| Initial visit | 18 | 0.0 | [0.0, 1.0] | (0.0, 2.0) |
| Final visit | 18 | 0.0 | [0.0, 1.0] | (0.0, 3.0) |
| Change | 18 | 0.0 | [0.0, 0.0] | (−2.0, +3.0) |
| Overall sleep quality | | | |  |
| Initial visit | 18 | 1.0 | [1.0, 2.0] | (0.0, 2.0) |
| Final visit | 16 | 1.0 | [0.5, 1.0] | (0.0, 2.0) |
| Change | 16 | 0.0 | [−1.0, 0.0] | (−1.0, +1.0) |
| Needs medications to sleep | | | |  |
| Initial visit | 18 | 0.0 | [0.0, 0.0] | (0.0, 3.0) |
| Final visit | 16 | 0.0 | [0.0, 0.0] | (0.0, 0.0) |
| Change | 16 | 0.0 | [0.0, 0.0] | (−3.0, 0.0) |
| PSQI total score | | | |  |
| Initial visit | 15 | 6.0 | [3.0, 7.0] | (3.0, 13.0) |
| Final visit | 15 | 4.0 | [3.0, 6.0] | (2.0, 8.0) |
| Change | 12 | −1.0 | [−2.0, 0.0] | (−7.0, +4.0) |

IQR: interquartile range; PSQI: Pittsburgh Sleep Quality Index.

PSQI sub-scores ranged from 0=best to 3=worst.

PSQI total score ranged from 0=best to 21=worst.

#### Safety

No new safety signals were identified. In the safety analysis set (n=30), 52 treatment-emergent adverse events (TEAEs) were documented in 23 patients. Twenty-eight TEAEs in 15 patients were considered drug-related (Most commonly dizziness or headache [n=4 each]). Three patients had a serious TEAE (cardiac failure, n=2; atrial fibrillation, n=1; none considered drug-related). One case of cardiac failure had a fatal outcome; the other 2 cases recovered. Five device events (most commonly device failure [n=2]) were documented in 4 patients; all were resolved.
